# Synthesis, Structural Studies and Antitumoral Evaluation of C-6 Alkyl and Alkenyl Side Chain Pyrimidine Derivatives

**DOI:** 10.3390/molecules14124866

**Published:** 2009-11-27

**Authors:** Svjetlana Krištafor, Tatjana Gazivoda Kraljević, Damjan Makuc, Janez Plavec, Lidija Šuman, Marijeta Kralj, Silvana Raić-Malić

**Affiliations:** 1 Department of Organic Chemistry, Faculty of Chemical Engineering and Technology, University of Zagreb, Marulićev trg 20, P.O. Box 177, HR-10000 Zagreb, Croatia; E-Mails: prekupec@fkit.hr (S.K.); tgazivod@fkit.hr (T.G.K.); 2 Slovenian NMR Centre, National Institute of Chemistry, Hajdrihova 19, P.O. Box 660, SI-1001 Ljubljana, Slovenia; E-Mails: damjan.makuc@ki.si (D.M.); janez.plavec@ki.si (J.P.); 3 Division of Molecular Medicine, Ruđer Bošković Institute, Bijenička 54, P.O. Box 1016, HR-10001, Zagreb, Croatia; E-Mails: lidija.suman@irb.hr (L.S.); marijeta.kralj@irb.hr (M.K.)

**Keywords:** C-6 alkyl and alkenyl pyrimidine derivatives, NMR conformational analysis, cytostatic activity evaluations

## Abstract

The synthetic route for introduction of fluorophenylalkyl (compounds **5**, **7**, **14** and **15**) and fluorophenylalkenyl (compounds **4E **and **13**) side chains at C-6 of the pyrimidine nucleus involved the lithiation of the pyrimidine derivatives **1**, **2** and **11** and subsequent nucleophilic addition or substitution reactions of the organolithium intermediate thus obtained with 2-fluorophenylacetone, 4-fluoroacetophenone or ethyl 4-fluorobenzoate as electrophiles. The structures of novel compounds were confirmed by ^1^H-, ^19^F- and ^13^C-NMR and MS. Compounds **8** and **10** containing unsaturated fluorophenylalkyl side chains showed better inhibitory effect than their saturated fluorophenylalkylated pyrimidine counterparts **7 **and **9**. A conformational study based on NOE enhancements showed the importance of the double bond and substitution in the side chain for the conformational preferences in relation to inhibitory activity. Among all tested compounds, C-5 furyl (**12**) and phenyl (**13 **and **15**) substituted pyrimidine derivatives showed significant cytostatic activities against all tested tumor cell lines.

## Introduction

The application of fluorine-containing compounds in the pharmaceutical and agrochemical fields has a very short history [1,2]. The small size of the fluorine substituent, combined with its high electronegativity and its impact upon bond strengths give rise to the observed distinctive effect of fluorine substituents on the biological activity of compounds [3]. In the area of medicinal chemistry, incorporation of fluorine has played a significant role in the development of new anti-cancer and anti-viral agents, anti-inflammatory and anti-hyperintensive agents, anti-fertility drugs and central nervous system drugs. Fluorine affects the biological activity of compounds in a number of important ways. It has been reported that covalently bonded fluorine is a very weak intermolecular hydrogen-bond acceptor [4]. The presence of fluorine at a particular position in a molecule can enhance its metabolic stability or modulate its physicochemical properties, such as its lipophilicity, acidity or basicity. Fluorination can increase molecules’ binding affinity to a target protein, and by a combination of factors interfere with specific enzyme action [2,5]. As a result, the introduction of fluorine atoms, trifluoromethyl, difluoromethyl or other fluorinated and fluoroalkyl groups into heterocyclic compounds may have significant influence on their biological and physical properties. Therefore, fluorinated compounds in general and fluorinated heterocycles in particular are the focus of much research [2]. Pyrimidines are biologically important molecules and valuable heterocyclic nuclei for the design of pharmaceutical agents [6,7]. A great number of C-5 and C-6 substituted pyrimidine nucleosides have been prepared in view of their various biological activities [8]. For this reason, the development of synthetic methods for fluorine-containing heterocyclic compounds has been an important field in both organofluorine chemistry and organic synthesis. 

Recently, we have reported that fluorinated propyl or propenyl C-6 acyclic pyrimidine derivatives containing the 2-hydroxy-3,3,3-trifluoro-1-propenyl side chain exhibited a pronounced effect against breast carcinoma (MCF-7), while the compound with a 2-fluoromethyl-2-acetoxypropyl chain exhibited moderate effect against cervical carcinoma (HeLa) [9]. We have also reported the synthesis and biological results of a new type of C-6 fluoroalkylated and fluorophenylalkylated pyrimidine derivatives as model compounds for development of tracer molecules in positron-emission tomography (PET) [10]. In this study we present the syntheses and antitumoral evaluations of C-6 fluorophenylalkyl (**6**, **9**, **14** and **15**), fluorophenylalkenyl (**4**, **8**, **10** and **13**) and 5-furyl-6-methyl (**12**) pyrimidine derivatives.

## Results and Discussion

### Chemistry

**Scheme 1:**
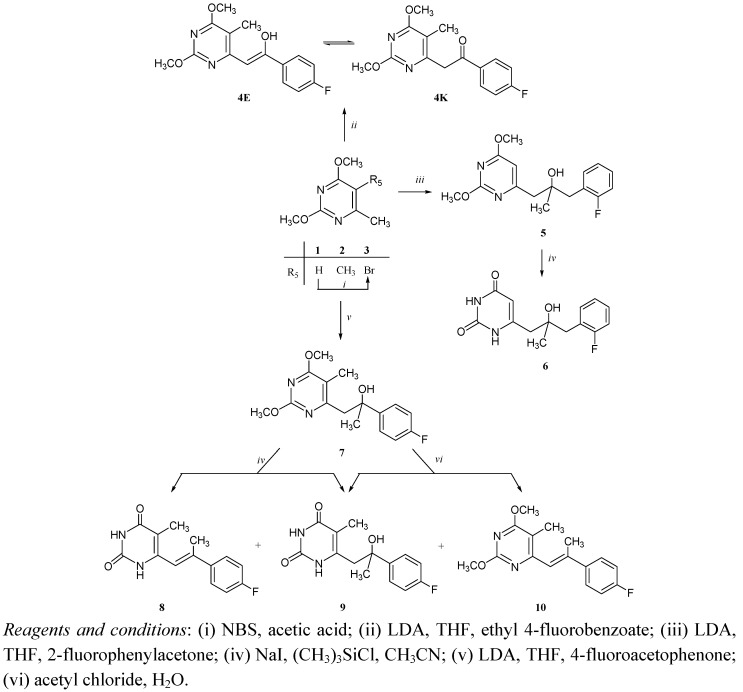
Synthesis of C-6 alkyl and alkenyl side chain pyrimidine derivatives (**4**-**10**).

**Scheme 2:**
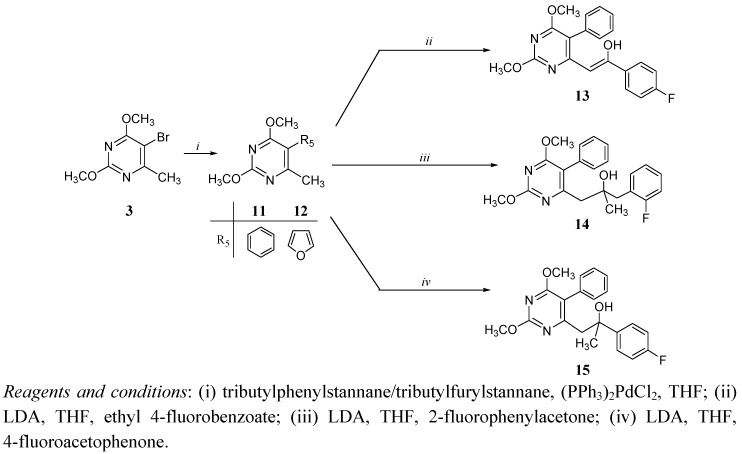
Synthesis of 5-phenylpyrimidine derivatives with C-6 fluorophenylalkyl side chain (**13**-**15**).

Key precursors 2,4-dimethoxy-6-methyl pyrimidine (**1**), 2,4-dimethoxy-5,6-dimethylpyrimidine (**2**) and 5-bromo-2,4-dimethoxy-6-methylpyrimidine (**3**) were prepared according to the procedure described in the literature [11,12]. The C-6 fluorophenylalkyl substituted pyrimidine derivatives **4**, **5**, **7** and **13**-**15** (Scheme 1, Scheme 2) were synthesized by lithiation, which has been studied extensively as an important carbon-carbon bond forming reaction [13,14]. Fluorophenyl-propyl and –ethyl side chaind were introduced by lithiation and subsequent nucleophilic addition of pyrimidine derivatives **1** and **2** to 2-fluorophenylacetone and 4-fluoroacetophenone to give **5** and **7**, respectively (Scheme 1). 

Reaction of lithiatied intermediate **2** with ethyl 4-fluorobenzoate afforded the C-6 substituted pyrimidine derivative containing unsaturated side chain, which exists as both the keto (**4K**) and enol (**4E**) tautomers (Scheme 1). The deprotection of 2,4-dimethoxypyrimidine derivative bearing C-6 fluorophenylalkyl side chain (**5**) with sodium iodide and trimethylchlorosilane gave pyrimidin-2,4-dione derivative **6**. Using the same synthetic procedure compound **7** yielded its unprotected pyrimidin-2,4-dione derivatives with unsaturated (**8**) and saturated (**9**) fluorophenylalkyl side chain. Reaction of **7 **with acetyl chloride and water gave deprotected pyrimidin-2,4-dione **9** and the 2,4-dimethoxypyrimidinederivative **10** with unsaturated side chain as a product of dehydration reaction. The reason for production of unsaturated derivatives **8** and **10 **is probably in enhanced stability of those compounds due to conjugated π-system of a pyrimidine and phenyl ring *via* side chain double bond, which is not present in compound **6** with the longer C-6 side chain.

The Stille reaction [15,16,17] was applied for the introduction of the phenyl and furyl rings at C-5 of pyrimidine moiety in **11** and **12**, respectively, using tributylphenylstannane and tributylfurylstannane with dichlorobis(triphenylphosphine)palladium(II) as a catalyst. C-6 alkylation of 5-furylpyrimidine derivative **12** did not give desired fluorophenylalkylated 5-furylpyrimidine product. Nucleophilic substitution and addition reactions of lithiated 5-phenylpyrimidine derivative **11** with corresponding electrophile as reagent afforded pyrimidine derivatives containing *p*-fluorophenyl-2-hydroxyethenyl (**13**), *o*-fluorobenzyl-2-hydroxypropyl (**14**) and *p*-fluorophenyl-2-hydroxypropyl (**15**) side chain (Scheme 2).

### Antitumoral activities

**Figure 1:**
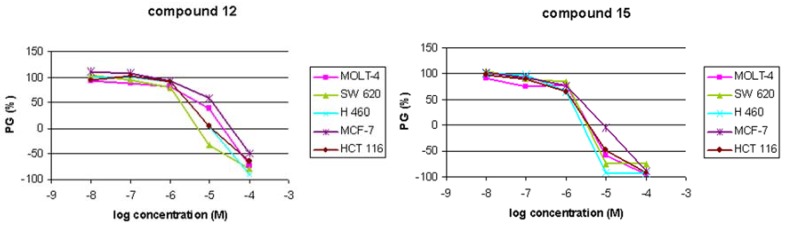
Dose-response profiles for compounds **12 **and **15**. PG = percentage of growth.

Compounds **4**, **6**, **8-10 **and **12**-**15 **were evaluated for their activities against human malignant tumor cell lines: Molt-4 (acute lymphoblastic leukemia), colon carcinoma (HCT 116 and SW 620), breast carcinoma (MCF-7) and lung carcinoma (H 460) (Table 1, Figure 1). 

The results point to a strong impact of methyl, phenyl, or furyl C-5 substituents. For example, 5-unsubstituted pyrimidine derivatives bearing the same C-6 fluoroalkyl side chain as their structurally related 5-substituted pyrimidine derivatives did not exhibit any cytostatic effect, as shown in our previous paper [10]. In addition*,* pyrimidine derivative with fluorophenylalkylated side chain **4** exhibited moderate cytotoxic activity against all malignant tumor cells tested, particularly against human breast carcinoma (MCF-7, IC_50_ = 2 µM). Furthermore, replacing of 5-methyl group (in **4** and **7**) with 5-phenyl moiety (in **13 **and **15**) improves the antiproliferative activity by an order of magnitude. Moreover, comparing the activities of 2,4-dimethoxy-6-methyl-5-phenylpyrimidine (**11**) [10] and 5-furyl-2,4-dimethoxy-6-methylpyrimidine (**12**) presented here (IC_50_ = 2 – 10 μM), it is obvious that replacing a phenyl with a furyl moiety strongly potentiates the antiproliferative activity. However, introduction of C-6 side chain in 5-phenylpyrimidine derivative **11** significantly increased the cytostatic effects of compounds **13**-**15**. Conformationally restricted compounds **8** (IC_50_ ≈ 69 µM) and **10** (IC_50_ ≈ 47 µM) containing C-6 unsaturated side chain showed higher antiproliferative effect than their analogues with saturated side chain **7** (IC_50_ > 100 µM) [10] and **9 **(IC_50_ > 100 µM). Deprotection of 2,4-diketo functionalities of **5** and **7** caused a loss of activity for **6** and **9**. 

In conclusion, from C-6 fluorophenylalkyl substituted 2,4-dimethoxy-5-phenylpyrimidines (**13**-**15**), compounds **13** (IC_50_ ≈ 2.5 µM) and **15** (IC_50_ ≈ 1.6 µM) containing a *p*-fluorophenyl moiety in the side chain showed the most pronounced activity. The effect of the 5-furylpyrimidine **12** and **15**, as the best representatives of the 5-phenylpyrimidine derivatives, were additionally tested on the cell cycle phases perturbation.

**Table 1:** Inhibitory effects of compounds **4**, **6**, **8-10** and **12**-**15** on the growth of human tumor cell lines.

Compd	IC_50_*^a^* (μM)				
	Molt-4	HCT 116	SW 620	MCF-7	H 460
**4**	43 ± 41	41 ± 10	32 ± 19	2 ± 1	19 ± 5
**6**	> 100	> 100	> 100	> 100	> 100
**8**	78 ± 21	79 ± 21	35 ± 6	51 ± 18	± 100
**9**	> 100	± 100	> 100	> 100	> 100
**10**	50 ± 45	37 ± 0.03	32 ± 10	42 ± 1	75 ± 3
**12**	6 ± 4	3 ± 0.3	2 ± 1.6	10 ± 6	4 ± 3
**13**	N.T. *^b^*	2 ± 0.2	2 ± 0.2	3 ± 1	3 ± 0.1
**14**	N.T. *^ b^*	14 ± 0.1	17 ± 0.1	14 ± 3	17 ± 2
**15**	2 ± 0.03	1 ± 0.5	2 ± 0.4	2 ± 1	1 ± 0.4

*^a^* IC_50_; 50% inhibitory concentration, or compound concentration required to inhibit tumor cell proliferation by 50%; *^b^* not tested.

### Cell cycle perturbations

**Figure 2:**
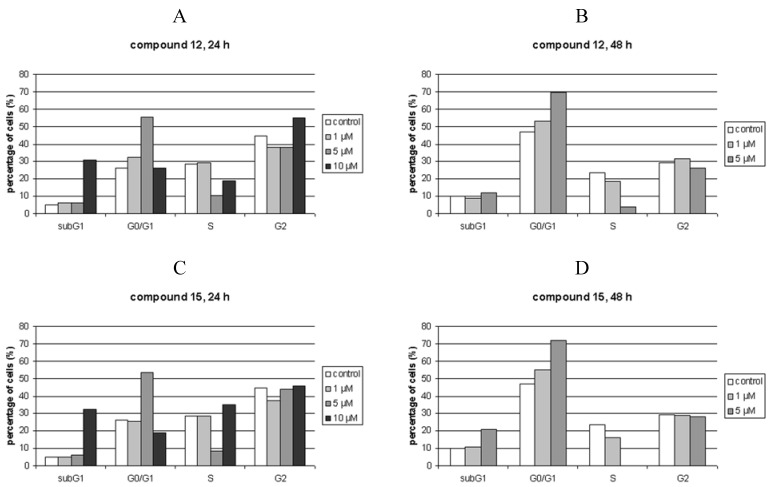
Cell cycle analysis of HCT 116 cells treated with 1, 5 and 10 μM compounds **12 **and **15**, 24 h (A and C, respectively), or 48 h (B and D, respectively). The histograms show percentages of live cells in G0/G1, S or G2/M phase, along with the number of dead (subG1) cells, where subG1 population is expressed as a percentage of total number of measured cells/counts.

Compounds **12** and **15** were tested at various concentrations close or slightly higher than their IC_50_ (1, 5 and 10 μM) on HCT 116 cells (Figure 2). Both compounds showed similar influence on the cell cycle, whereby strong accumulation of cells in G1 cell cycle phase accompanied with drastic reduction of cells in S phase was detected. These effects were strongly dose-dependent. Moreover, compound **15** dose-dependently increased the percentage of cells in sub G1 after 48 hours of treatment, pointing to the induction of apoptosis. In addition, both compounds at 10 μM concentration induced cell death of more than 30% of cells after 24 hours, while all cells were dead after 48 hours (not shown).

These results unambiguously point to the effects of here presented compounds on the cell cycle phenomena that occur probably during G1 phase and inhibit DNA synthesis, i.e. inhibit the progression of cell to next cell cycle phase. Being unable to progress through S phase to mitosis (M phase), the cells die, most probably by apoptosis.

### Structural and conformational studies by NMR

^1^H-, ^19^F- and ^13^C-NMR data given in Table 2 and in the Experimental section are in full agreement with the structures of **4E**, **4K**, **6**, **8**-**10**, **13 **and **14**. The observed chemical shifts of ^1^H- and ^13^C- resonances are characteristic of a C-6 substituted pyrimidine moiety.

**Table 2:** ^1^H- and ^19^F-NMR chemical shifts (δ/ppm), signal multiplicities and H-H coupling constants (*J*/Hz).

Compd	N1–H	N3–H	H5	H1'	C2'–OH	C2'–Me	C5–Me	–OCH_3_	phenyl	^19^F
**4E***^a^*	–	–	–	6.29 (s)	15.08 (s)	–	2.09 (s)	3.95 (s)	7.97 (m, Hϕ2/Hϕ6)	–110.31 (m)
									7.29 (m, Hϕ3/Hϕ5)	
**4K***^a^*	–	–	–	4.48 (s)	–	–	1.97 (s)	3.75 (s), 3.91 (s)	8.10 (m, Hϕ2/Hϕ6)	–105.21 (m)
									7.37 (m, Hϕ3/Hϕ5)	
**6***^b^*	10.92 (s)	10.33 (s)	5.37 (d)	2.46 (d)	4.73 (s)	1.02 (s)	–	–	7.14 (m, Hϕ3)	–115.83 (m)
									7.27 (m, Hϕ4)	
									7.14 (m, Hϕ5)	
									7.33 (m, Hϕ6)	
**8***^c^*	11.06 (s)	10.59 (s)	–	6.39 (m)	–	2.02 (d)	1.70 (d)	–	7.64 (m, Hϕ2/Hϕ6)	–113.36 (m)
									7.24 (m, Hϕ3/Hϕ5)	
**9***^d^*	10.93 (s)	10.33 (s)	–	2.73 (d) 2.82 (d)	5.52 (b)	1.52 (s)	1.50 (s)	–	7.51 (m, Hϕ2/Hϕ6)	–
									7.13 (m, Hϕ3/Hϕ5)	
**10***^e^*	–	–	–	6.78 (q)	–	2.43 (d)	2.06 (s)	3.89 (s), 3.92 (s)	7.66 (m, Hϕ2/Hϕ6)	–113.79 (m)
									7.24 (m, Hϕ3/Hϕ5)	
**13**	–	–	–	6.93 (s)	11.67 (s)	–	–	3.79 (s)	7.40 (m, Hϕ2/Hϕ6)	–
									7.33 (m, Hϕ3/Hϕ5)	
								3.90 (s)		
									7.22 (m, 5Hϕ)	
**14***^f^*	–	–	–	2.61 (s)	5.19 (s)	0.95 (s)	–	3.80 (s)	7.17 (m, Hϕ3)	–
									7.31 (m, Hϕ4)	
									7.19 (m, Hϕ5)	
								3.92 (s)		
									7.38 (m, Hϕ6)	
									7.02 (m, 5Hϕ)	

^a^ The 4K : 4E ratio is ca. 55 : 45 as observed in ^1^H and ^19^F NMR spectra; ^b^^4^*J*_H1’-H5_ = 1.5, multiplet for H3': 2.74 (d), 2.79 (d) with ^2^*J*_H gem_ = 13.4 Hz; ^c^^4^*J*_H1'-C2'Me_ = 1.2 and ^5^*J*_H1'-C5Me_ = 0.6 Hz; ^d^ ^2^*J*_H1' gem_ = 13.9 Hz; ^e^^4^*J*_H1'-C2'Me_ = 1.2 Hz; ^f^ Multiplet for H3': 2.79 (d), 2.68 (d) with ^2^*J*_H gem_ = 13.7 Hz.

**Figure 3:**
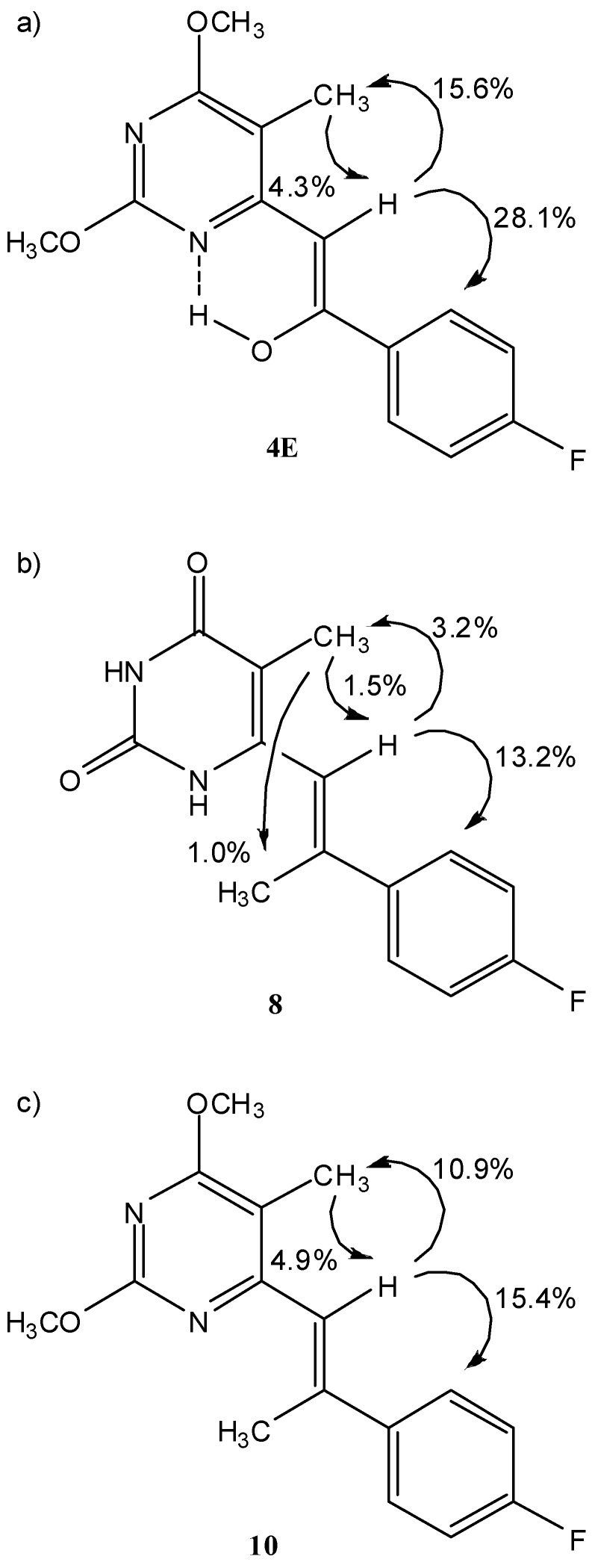
The key NOE enhancements and configuration along C1'=C2' double bond for a) **4E**, b) **8** and c) **10 **in DMSO-*d*_6_ solution.

The conformational properties of **4E**, **8 **and **10** and the configuration along C1'=C2' double bond were assessed with the use of 1D difference NOE experiments. The saturation of H1' (*δ* 6.29) in **4E** resulted in strong NOE enhancements at C5-Me and ϕ2/ϕ6-protons which confirmed the *Z*-configuration along C1'=C2' double bond (Figure 3a). In addition, the saturation of C5-Me protons (*δ* 2.09) resulted in NOE at H1'. The observed NOE enhancements are in agreement with a conformation in which C2'-OH group is predisposed for the formation of the N1···HO hydrogen bond as shown in Figure 3a. In fact, strongly deshielded ^1^H-NMR signal at *δ* 15.08 ppm corresponding to hydroxyl group supports its involvement in hydrogen bond (Table 2). 

The saturation of H1' (*δ* 6.39) in **8** showed strong NOE enhancements at ϕ2/ϕ6-protons of fluorinated phenyl ring and none at C2'-Me which is in agreement with *E*-configuration along C1'=C2' double bond. On the other hand, the saturation of H1' resulted in a weak NOE enhancement at C5-Me group (Figure 3b). The saturation of C5-Me protons (*δ* 1.70) showed weak NOE enhancements at both H1' and C2'-Me group which suggested conformational freedom along C6-C1' single bond (Figure 3b).

The saturation of H1' (*δ* 6.78) in **10** resulted in strong NOE enhancements at C5-Me and ϕ2/ϕ6-protons of fluorinated phenyl ring (Figure 3c). The absence of NOEs with C2'-Me protons confirmed *E*-configuration along C1'=C2' double bond as shown in Figure 1c. Furthermore, the saturation of C5-Me protons (*δ* 2.06) showed NOE enhancement at H1'. These results are in agreement with the predominant conformation in which H1’ is spatially closer to the C5-Me group.

## Experimental

### General

Melting points (uncorrected) were determined on a Kofler micro hot-stage (Reichert, Wien). Precoated Merck silica gel 60F-254 plates were used for thin layer chromatography (TLC) and the spots were detected under UV light (254 nm). Column chromatography (CLC) was performed using silica gel (0.063-0.2 mm) Fluka; glass column was slurry-packed under gravity. The electron impact mass spectra were recorded with an EXTREL FT MS 2001 instrument with ionizing energy 70 eV. Elemental analyses were performed in the Central Analytic Service, Ruđer Bošković Institute, Zagreb. ^1^H-, ^13^C- and ^19^F-NMR spectra were acquired on a Varian Unity Inova 300 MHz NMR spectrometer. All data were recorded in DMSO-*d*_6_ at 298 K. Chemical shifts were referenced to the residual solvent signal of DMSO at δ 2.50 ppm for ^1^H and *δ* 39.50 ppm for ^13^C, whereas fluorine chemical shifts were referenced relative to external CCl_3_F (*δ* 0.0 ppm). Individual resonances were assigned on the basis of their chemical shifts, signal intensities, multiplicity of resonances, H-H coupling constants involved as well as with the use of a series of 2D NMR experiments (gHSQC and gHMBC).

### Procedures for the preparation of compounds

2,4-Dimethoxy-6-methylpyrimidine (**1**), 2,4-dimethoxy-5,6-dimethylpyrimidine (**2**), 2,4-di-methoxy-6-methyl-5-bromopyrimidine (**3**), 6-[2-(2’-fluorobenzyl)-2-hydroxypropyl]-2,4-dimethoxy-pyrimidine (**5**), 6-[2-(4’-fluorophenyl)-2-hydroxypropyl]-2,4-dimethoxy-5-methyl-pyrimidine (**7**), 2,4-dimethoxy-6-methyl-5-phenylpyrimidine (**11**), 5-furyl-2,4-dimethoxy-6-methylpyrimidine (**12**) and 6-[2-(4’-fluorophenyl)-2-hydroxypropyl]-5-phenyl-2,4-dimethoxypyrimidine (**15**) were synthesized in accord with original procedures given in the literature [10,11,12,18].

### 6-[2-(4’-Fluorophenyl)-2-hydroxyethenyl]-2,4-dimethoxy-5-methylpyrimidine *(**4**)*

The solution of 2,4-dimethoxy-5,6-dimethylpyrimidine (**2**) (0.66 g, 3.94 mmol) in anhydrous THF (10 mL) was cooled at −70 °C and lithium diisopropylamide (LDA, 2.91 mL, 2 M in THF/heptane/ethylbenzene) was added dropwise to the reaction mixture. The temperature was then raised to −55 °C and the reaction mixture was stirred for 30 min. Ethyl 4-fluorobenzoate (0.69 mL, 4.73 mmol) was added and the mixture was additionally stirred for 3 h and then neutralized with glacial acetic acid. The temperature was raised to room temperature and the reaction mixture was stirred for an additional 15 min. The solvent was evaporated and the residual yellow oily product was extracted with CH_2_Cl_2_ and water. The organic layer was dried over Na_2_SO_4_ and purified by column chromatography using petroleum ether-ethyl acetate = 7:1 as eluent. After column chromatography, compound **4 **was isolated as a mixture of **4E** and **4K**. Yield = 0.28 g (24.7%); mp = 85-88 °C.

**4E: **^13^C-NMR (DMSO-*d*_6_) δ: 168.84 (C4), 163.24 (Cϕ4, d, ^1^*J*_CF _= 247.8), 163.13 (C2', d, ^5^*J*_CF _= 0.6), 162.39 (C6), 160.28 (C2), 131.49 (Cϕ1, d, ^4^*J*_CF _= 3.0), 128.84 (Cϕ2 and Cϕ6, d, ^3^*J*_CF _= 8.5), 115.46 (Cϕ3 and Cϕ5, d, ^2^*J*_CF _= 21.7), 102.74 (C5), 90.66 (C1', d, ^6^*J*_CF _= 1.4), 54.38 (C2-OCH_3_), 54.08 (C4-OCH_3_), 8.98 (C5-CH_3_).

**4K: **^13^C-NMR (DMSO-*d*_6_) δ: 194.57 (C2'), 169.09 (C4), 165.11 (Cϕ4, d, ^1^*J*_CF _= 251.6), 163.19 (C6), 162.14 (C2), 133.12 (Cϕ1, d, ^4^*J*_CF _= 3.3), 131.39 (Cϕ2 and Cϕ6, d, ^3^*J*_CF _= 9.6), 115.73 (Cϕ3 and Cϕ5, d, ^2^*J*_CF _= 22.0), 109.08 (C5), 54.01 (C2-OCH_3_), 53.95 (C4-OCH_3_), 45.00 (C1'), 9.75 (C5-CH_3_); MS *m/z* 291 (MH)^+^; Anal. calcd. for C_15_H_15_FN_2_O_3_: C, 62.06; H, 5.21; N, 9.65. Found: C, 61.94; H, 5.22; N, 9.69.

### 6-[2-(2’-Fluorobenzyl)-2-hydroxypropyl]pyrimidin-2,4-dione *(**6**)*

A mixture of compound **5** (0.08 g, 0.27 mmol), chlorotrimethylsilane (0.12 mL, 0.95 mmol) and NaI (0.14 g, 0.95 mmol) in anhydrous acetonitrile (6 mL) was stirred at room temperature for 18 h and then at 60 °C for 3 h under argon atmosphere. The solvent was evaporated under reduced pressure, and the residue was chromatographed using dichloromethane-methanol = 25:1 as eluent to give **6**. Yield = 50 mg (66.4%); mp = 157-159 °C. ^13^C-NMR (DMSO-*d*_6_) δ: 163.97 (C4), 160.91 (Cϕ2, d, ^1^*J*_CF _= 243.4), 152.95 (C6), 151.32 (C2), 133.38 (Cϕ6, d, ^3^*J*_CF _= 5.0), 128.32 (Cϕ4, d, ^3^*J*_CF _= 8.2), 124.25 (Cϕ1, d, ^2^*J*_CF _= 15.9), 123.78 (Cϕ5, d, ^4^*J*_CF _= 3.3), 114.88 (Cϕ3, d, ^2^*J*_CF _= 23.1), 100.81 (C5), 71.52 (C2', d, ^4^*J*_CF _= 1.1), 43.66 (C1', d, ^5^*J*_CF _= 0.6), 40.86 (C3', d, ^3^*J*_CF _= 5.5), 25.13 (C2'-CH_3_, d, ^5^*J*_CF _= 1.4); MS *m/z* 277 (M-H); Anal. calcd. for C_14_H_15_FN_2_O_3_: C, 60.42; H, 5.43; N, 10.07. Found: C, 60.61; H, 5.41; N, 10.11.

### 6-[2-(4’-Fluorophenyl)-1-propenyl]-5-methylpyrimidin-2,4-dione *(**8**)* and 6-[2-(4’-fluorophenyl)-2-hydroxypropyl]-5-methylpyrimidin-2,4-dione *(**9**)*

The deprotection of **7** was performed in the same manner as described for **6**. Reagents used were: compound **7** (0.26 g, 0.84 mmol), chlorotrimethylsilane (0.37 mL, 0.84 mmol) and NaI (0.44 g, 2.94 mmol) in anhydrous acetonitrile (7 mL). After column chromatography using dichloromethane-methanol = 40:1 as eluent crystalline compounds **8** and **9 **were isolated. **8**: Yield = 50 mg (24.4%); mp = 140-142 °C; **9**: Yield = 60 mg (27.3%); mp = 192–194 °C.

**8**: ^13^C-NMR (DMSO-*d*_6_) δ: 164.89 (C4), 162.14 (Cϕ4, d, ^1^*J*_CF _= 245.0), 150.79 (C2), 145.92 (C6), 141.77 (C2'), 137.08 (Cϕ1, d, ^4^*J*_CF _= 3.3), 128.09 (Cϕ2 and Cϕ6, d, ^3^*J*_CF _= 8.2), 118.05 (C1', d, ^6^*J*_CF _= 1.1), 115.28 (Cϕ3 and Cϕ5, d, ^2^*J*_CF _= 21.4), 105.55 (C5), 17.79 (C2'-CH_3_), 10.85 (C5-CH_3_); MS *m/z* 261 [MH]^+^; Anal. calcd. for C_14_H_13_F_2_N_2_O_2_: C, 64.61; H, 5.03; N, 10.76. Found: C, 64.48; H, 5.04; N, 10.78.

**9**: ^13^C-NMR (DMSO-d_6_) δ: 165.34 (C4), 161.42 (Cϕ4, d, ^1^*J_CF_* = 242.4), 150.84 (C2), 147.54 (C6), 144.63 (Cϕ1, d, ^4^*J_CF_* = 2.6), 127.42 (Cϕ2 and Cϕ6, d, ^3^*J_CF_* = 8.0), 114.84 (Cϕ3 and Cϕ5, d, ^2^*J_CF_* = 21.0), 107.22 (C5), 74.14 (C2'), 43.97 (C1'), 29.46 (C2’-CH_3_), 10.64 (C5-CH_3_); MS *m/z* 277 [M-H]; Anal. calcd. for C_14_H_15_FN_2_O_3_: C, 60.42; H, 5.43; N, 10.07. Found: C, 60.30; H, 5.45; N, 10.09.

### 6-[2-(4’-Fluorophenyl)-2-hydroxypropyl]-5-methylpyrimidin-2,4-dione *(**9**)* and 6-[2-(4’-fluorophenyl)-1-propenyl]-2,4-dimethoxy-5-methylpyrimidine *(**10**)*

Compound **7** (0.16 g, 0.52 mmol) was dissolved in acetyl chloride (7 mL) and the reaction mixture was refluxed for 5 h. Water (2 mL) was then added and the reaction mixture was stirred overnight at room temperature. The solvent was evaporated at the reduced pressure and the remaining yellow oil was chromatographed using dichloromethane as eluent, which yielded compounds **9** and **10**. **9**: Yield = 60 mg (44.3%); **10**: Yield = 20 mg (9.8%); mp = 66–68 °C.

**10**: ^13^C-NMR (DMSO-*d*_6_) δ: 169.52 (C4), 162.01 (Cϕ4, d, ^1^*J*_CF _= 245.0), 162.0 (C6), 162.0 (C2), 143.03 (C2'), 139.22 (Cϕ1, d, ^4^*J*_CF _= 3.0), 128.20 (Cϕ2 and Cϕ6, d, ^3^*J*_CF _= 8.2), 122.11 (C1', d, ^6^*J*_CF _= 1.7), 115.24 (Cϕ3 and Cϕ5, d, ^2^*J*_CF _= 21.4), 108.42 (C5), 54.10 (C2-OCH_3_), 53.92 (C4-OCH_3_), 17.85 (C2'-CH_3_), 9.89 (C5-CH_3_); MS *m/z* 289 [MH]^+^; Anal. calcd. for C_16_H_17_FN_2_O_2_: C, 66.65; H, 5.94; N, 9.72. Found: C, 66.92; H, 5.93; N, 9.74.

### 6-[2-(4’-Fluorophenyl)-2-hydroxy-1-ethenyl]-2,4-dimethoxy-5-phenylpyrimidine *(**13**)*

The synthesis of **13** was performed in the same manner as described for **4**. Reagents used were: compound **11 **(138 mg, 0.59 mmol), LDA (0.58 mL, 2 M in THF/heptane/ethylbenzene) and ethyl 4-fluorobenzoate (0.09 mL, 0.58 mmol) in anhydrous THF (6 mL). After column chromatography using cyclohexane-ethyl acetate = 6:1 as eluent compound **13** was isolated as crude oil. Yield = 44.3 mg (16.6%). ^13^C-NMR (DMSO-*d*_6_) δ: 167.99 (C4), 163.55 (Cϕ4, d, ^1^*J*_CF _= 233.5), 163.03 (C2'), 162.35 (C6), 160.52 (C2), 134.00 (Cϕ1, d, ^4^*J*_CF _= 2.7), 132.88 (Cϕ1'), 131.08 (Cϕ2 and Cϕ6, d, ^3^*J*_CF _= 8.8), 130.79 (Cϕ2'), 129.12 (Cϕ3'), 128.00 (Cϕ4'), 127.87 (Cϕ3 and Cϕ5, d, ^2^*J*_CF _= 18.3), 122.33 (C1'), 114.08 (C5), 54.79 (C2-OCH_3_), 54.55 (C4-OCH_3_); MS *m/z* 353 [MH]^+^; Anal. calcd. for C_20_H_17_FN_2_O_3_: C, 68.17; H, 4.86; N, 7.95. Found: C, 68.37; H, 4.84; N, 7.97.

### 6-[2-(2’-Fluorobenzyl)-2-hydroxypropyl]-2,4-dimethoxy-5-phenylpyrimidine *(**14**)*

According to the procedure described for **4**, the synthesis of **14** was performed using compound **11 **(95 mg, 0.41 mmol), LDA (0.40 mL, 2 M in THF/heptane/ethylbenzene) and 2-fluorophenylacetone (0.09 mL, 0.41 mmol) in anhydrous THF (6 mL) as reagents. After column chromatography using petroleum ether-ethyl acetate = 6:1 as eluent compound **14** was isolated. Yield = 40.3 mg (25.7%). ^13^C-NMR (DMSO-*d*_6_) δ: 169.19 (C4), 161.95 (Cϕ2, d, ^1^*J*_CF _= 245.7), 166.56 (C6), 163.02 (C2), 133.91 (Cϕ6, d, ^3^*J*_CF _= 4.7), 133.54 (Cϕ1’), 131.25 (Cϕ2’), 130.90 (Cϕ4, d, ^3^*J*_CF _= 7.2), 129.58 (Cϕ3’), 125.16 (Cϕ4’), 116.24 (Cϕ1, d, ^2^*J*_CF _= 14.0), 123.77 (Cϕ5, d, ^4^*J*_CF _= 3.3), 115.00 (Cϕ3, d, ^2^*J*_CF _= 22.7), 116.25 (C5), 72.91 (C2'), 44.28 (C1'), 54.89 (C2-OCH_3_), 54.51 (C4-OCH_3_). 40.85 (C3', d, ^3^*J*_CF _= 5.6), 27.14 (C2'-CH_3_); MS *m/z* 383 [MH]^+^; Anal. calcd. for C_22_H_23_FN_2_O_3_: C, 69.09; H, 6.06; N, 7.33. Found: C, 69.29; H, 6.04; N, 7.31.

### Antitumor activity assays

The colon carcinoma (HCT 116 and SW 620), breast carcinoma (MCF-7) and lung carcinoma (H 460) cells were cultured as monolayers and maintained in Dulbecco's modified Eagle's medium (DMEM), while MOLT-4 cells (acute lymphoblastic leukemia) were cultured in suspension in RPMI medium, both supplemented with 10% fetal bovine serum (FBS), 2 mM L-glutamine, 100 U/mL penicillin and 100 μg/mL streptomycin in a humidified atmosphere with 5% CO_2_ at 37 °C.

The growth inhibition activity was assessed as described previously, according to the slightly modified procedure of the National Cancer Institute, Developmental Therapeutics Program [9,10]. Briefly, the cells were inoculated onto standard 96-well microtiter plates on day 0. Test agents were then added in five consecutive 10-fold dilutions (10^-8^ to 10^-4^ mol/L) and incubated for further 72 hours. Working dilutions were freshly prepared on the day of testing. The solvent (DMSO) was also tested for eventual inhibitory activity by adjusting its concentration to be the same as in working concentrations (maximal concentration of DMSO was 0.25%). After 72 hours of incubation, the cell growth rate was evaluated by performing the MTT assay [10] which detects dehydrogenase activity in viable cells. The absorbency (OD, optical density) was measured on a microplate reader at 570 nm. 

Each test point was performed in quadruplicate in three individual experiments. The results are expressed as IC_50_, which is the concentration necessary for 50% of inhibition. The IC_50_ values for each compound are calculated from dose-response curves using linear regression analysis by fitting the test concentrations that give PG (percentage of growth) values above and below the reference value (i.e. 50%). Each result is a mean value from three separate experiments.

### Cell cycle analysis

2 × 10^5 ^cells were seeded per well in a 6-well plate. After overnight incubation, tested compounds were added. After desired length of time, the attached cells were trypsinized, combined with floating cells, washed with phosphate buffered saline (PBS) and fixed with 70% ethanol. Immediately before the analysis, cells were washed with PBS and incubated with 0.1 μg/μL RNAse A at 37 °C for 15 minutes. Subsequently, cells were stained with 50 µg/ml of propidium iodide (PI) and analyzed by Becton Dickinson FACScalibur flow cytometer. For each analysis, 20,000 events were measured. Measurements were performed in duplicate for two independent experiments. The percentage of the cells in each cell cycle phase was based on the obtained DNA histograms and determined using the ModFit LT^TM^ software (Varity House). Statistical analysis was performed in Microsoft Excel by using the ANOVA at p < 0.05.

## Conclusions

C-6 fluorophenylalkyl (**6**, **9**, **14** and **15**), fluoropenylakenyl (**4**, **8**, **10** and **13**) and 5-furyl-6-methyl (**12**) pyrimidine derivatives were synthesized and evaluated for their cytostatic activities. Among all tested compounds, C-5 furyl (**12**) and phenyl (**13 **and **15**) substituted pyrimidine derivatives showed the most significant cytostatic activities against all tumor cell lines (IC_50_ are in low micromolar range). In the series of C-6 fluorophenylalkyl 5-methylpyrimidines, compounds **8** and **10** containing unsaturated side chains showed better inhibitory effects (**8**: IC_50_ ≈ 69 µM, **10**: IC_50_ ≈ 47 µM) than their saturated pyrimidine derivatives **7** and **9**. Furthermore, among C-6 fluorophenylalkyl 5-phenyl-pyrimidines, compounds **13** and **15 **with a *p*-fluorophenyl moiety in the side chain exhibited the most pronounced antiproliferative activity (**13**: IC_50_ ≈ 2.5 µM, **15**: IC_50_ ≈ 1.6 µM). Besides, **12** and **15** induced strong changes in the cell cycle of tumor cells (accumulation of cells in G1 phase and drastic decrease in the number of cells in S phase), which eventually caused death of tumor cells. Thus, these compounds emerged as the most interesting leading compounds that could be used for further structural optimization. 
